# Marine Bacteria under Low-Intensity Radioactive Exposure: Model Experiments

**DOI:** 10.3390/ijms24010410

**Published:** 2022-12-27

**Authors:** Olga V. Kolesnik, Tatiana V. Rozhko, Nadezhda S. Kudryasheva

**Affiliations:** 1 Institute of Biophysics SB RAS, Federal Research Center ‘Krasnoyarsk Science Center SB RAS’, 660036 Krasnoyarsk, Russia; 2 Biophysics Department, Siberian Federal University, 660041 Krasnoyarsk, Russia; 3 FSBEI HE V.F. Voino-Yasenetsky KrasSMU MOH, 660022 Krasnoyarsk, Russia

**Keywords:** low-dose, radionuclides, radiotoxicity, radiation hormesis, bioassay, luminous bacteria, enzymes, radioprotection, humic substances

## Abstract

Radioactive contaminants create problems all over world, involving marine ecosystems, with their ecological importance increasing in the future. The review focuses on bioeffects of a series of alpha and beta emitting radioisotopes (americium-241, uranium-(235 + 238), thorium-232, and tritium) and gamma radiation. Low-intensity exposures are under special consideration. Great attention has been paid to luminous marine bacteria as representatives of marine microorganisms and a conventional bioassay system. This bioassay uses bacterial bioluminescence intensity as the main testing physiological parameter; currently, it is widely applied due to its simplicity and sensitivity. Dependences of the bacterial luminescence response on the exposure time and irradiation intensity were reviewed, and applicability of hormetic or threshold models was discussed. A number of aspects of molecular intracellular processes under exposure to low-intensity radiation were analyzed: (a) changes in the rates of enzymatic processes in bacteria with the bioluminescent system of coupled enzymatic reactions of NADH:FMN-oxidoreductase and bacterial luciferase taken as an example; (b) consumption of an intracellular reducer, NADH; (c) active role of reactive oxygen species; (d) repairing of the DNA damage. The results presented confirm the function of humic substances as natural radioprotectors.

## 1. Introduction

Nowadays, low-intensity radioactive contaminants create problems all over the world. A tentative limit of low-dose effects for higher organisms is supposed to be 0.1 Gy, according to ICRP Publication 99 [1]. Generally, specialists in radiotoxicology suggest that this border separates low-dose and toxic radiation effects; the latter means the suppression of physiological functions of organisms under radioactive exposure. However, the data accumulated show that this border can change under varied environmental conditions, as well as the type and the state of an organism [2,3].

The biological effects of low-intensity radiation are currently of particular interest due to the permanent expansion of contaminated areas. Radioactive decay products can affect the associated chemical and biological processes in natural ecosystems, accompanied by a natural change in the balance of the ecosystem as a whole. To identify molecular mechanisms of the low-dose radiation bioeffects, proper bioindicators should be chosen and adapted.

Molecular mechanisms of biological responses to low-dose radiation form a basis for predicting responses of organisms to low-intensity radiation due to discharges of nuclear plants, underground mining, or nuclear accidents. Thus, these mechanisms are of practical interest.

Unicellular organisms are proper tools for studying biological responses in complex media, being the initial link of food chains in aquatic ecosystems, and hence, defining the state of the ecosystem as a whole [4,5]. Physiological parameters of unicellular organisms can serve as indicators of the state of the ecosystem. Therefore, microorganisms can be used to monitor environmental toxicity, including radiotoxicity [6]. Radiation effects have only been investigated in a small subset of microbial species [7,8], and studies involving other species can contribute to understanding the biological effects of radiation.

Bioassays based on luminous marine bacteria are suitable candidates for monitoring environmental toxicity. These bioassays use bacterial luminescence as a physiological function in test procedures and can be used to study both chronic and acute radioactive exposures. Due to high sensitivity to toxic compounds, luminous marine bacteria have been used as a toxicity biotest for more than fifty years [6,9,10,11,12]. Bioluminescence assays are similar to spectrophotometric tests in their simplicity and high rate of test procedures.

The bacterial bioluminescent enzyme system was suggested for the first time in 1990 [13]. This is another type of bioluminescent assay. Advantages of the enzymatic assay include a possibility to change sensitivity to toxicants by varying the component concentration and composition as well as by constructing coupled polyenzymatic systems [14,15,16,17]. Technological applications of the bioluminescent enzymatic system were reviewed in [18,19].

The conventional bioluminescent enzymatic assay is based on the coupled bacterial bioluminescent enzyme system; it involves two enzymatic reactions. The first one, catalyzed by NADH:FMN-oxidoreductase, is the reduction of flavin mononucleotide (FMN) by nicotinamide adenine dinucleotide (NADH):

$$FMN+NADH\overset{NADH:FMN-oxidoreductase}{\to }FMN\cdot H^{-}+NADH^{+}$$

In the second reaction, catalyzed by bacterial luciferase, the reduced flavin (ionized form) and long-chain aldehyde are oxidized by molecular oxygen, to yield the corresponding acid, H_2_O, FMN, and a quantum of light (λ_max_ about 500 nm):

$$FMN\cdot H^{-}+RCHO+O_{2}\overset{luciferase}{\to }FMN+RCOO^{-}+H_{2}O+hv$$

Interference with any part of the bacterial cell metabolism that affects the release of components of the bacterial bioluminescence will be indicated by a decrease in the light emission. Based on the extensive investigation of effects of model toxic exogenous compounds, a classification of the effects on the bioluminescent enzymatic assay system was suggested [20] and developed. The effects of different groups of exogenous compounds, i.e., organic dyes [21,22,23], oxidizers [24,25,26], halogen-substituted molecules [27,28,29], and salts of stable and radioactive metals [16,29,30,31,32,33,34,35], were discussed according to the classification suggested.

The main advantage of the luminescence bioassays is a high rate of registration of the physiological function, luminescence intensity. This advantage provides a possibility to conduct a lot of experiments under comparable conditions and leads to adequate statistical processing. This is highly important for biological assays that are usually characterized by lower reproducibility as compared to chemical assays or radiometric measurements. This feature is particularly important in toxicological studies of low-dose effects, which are usually noisy and stochastic. 

It is known that biological responses to low-dose radiation may change upon variations in the molecular surrounding [2,3]. The addition of organic poly-functional molecules can change radical and ionic states of aqueous solutions of alpha- and beta-emitting radionuclides, thus changing the environment of water inhabitants, and therefore, their responses in radionuclide solutions. Aquatic biota might be sensitive to the presence of humic substances (HS), products of oxidative decomposition of organic matter in water body sediments, and natural complexing and redox agents, which play an important role as natural toxicity attenuators [36,37,38,39,40]. Phenolic and other active groups in HS result in their capability of mitigating toxic impacts of oxidizers of both organic and inorganic types [30,41,42], similarly to other natural phenolic compounds [43,44]. The biological activity of HS is currently discussed intensively [6,45,46,47,48,49,50].

This review is aimed at considering the bioeffects of radionuclides of alpha and beta types, such as thorium-232, americium-241, and uranium-(235 + 238), tritium, as well as gamma-irradiation, under the conditions of low-intensity irradiation. Luminous marine bacteria are chosen as an example of the model unicellular organism. A number of types of molecular mechanisms of responses to low-dose exposures are under discussion. Special attention is paid to the radioprotective effects of humic substances, natural attenuators of radiotoxicity, in the model solutions of radionuclides.

## 2. Low-Dose Radiation Effects on Luminous Marine Bacteria

Studies of the biological effects of low-dose exposures have been conducted since the 1960s [51]. The achievements of Calabrese (Laboratory of Toxicology, University of Massachusetts Settlement, USA) are widely known. [52,53]. The works of E.B. Burlakova’s group (Emanuel Institute of Biochemical Physics, Russia) are considered to be classical in the study of low-intensity exposures [54]. Nonlinear dose-effect relationships, commonly referred to as ‘hormesis’, have been described and discussed for low-dose exposure. This term was introduced in 1943 [55]. In general, this term refers to a positive biological response to low-dose exposure to toxicants or other stress-factors, including ionizing radiation. It is assumed that the phenomenon of hormesis is characteristic of all biological systems, regardless of the level of biological organization (cell, organ, and organism) and methods of registration. The first book on radiation hormesis was written by Luckey [56]. The intensity of the study of radiobiological low-dose effects has been increasing since the 1970s [54,57,58,59,60], including studies on microorganisms [61,62,63,64]. Thus far, two models have been investigated to describe the mechanism of radiation hormesis: this phenomenon is associated with either DNA damage or membrane processes [54,59,61,65,66,67,68,69].

**Figure 1:**
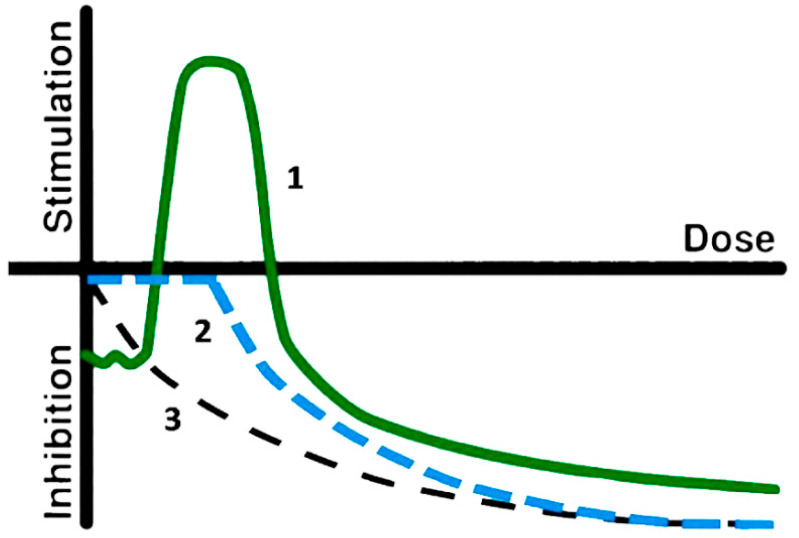
Scheme of the dose-effect models; (1) hormesis; (2) threshold; (3) linear [72].

In general, there exist three models of biological response to low-dose exposure. The first model, hormesis, is marked with the number 1 in Figure 1. Hormesis is the most complex dependence, and it is supposed to be the basic model; two other dependences, threshold (2), and linear no-threshold models, LNT (3), can be considered as particular cases of hormesis [70,71]. 

An integral element of the LNT hypothesis is the assumption that the nature of the biological response to radiation damage is linear, regardless of the dose. However, exposure of cells or organisms to low dose/dose-rate ionizing radiation elicits an adaptive response, resulting in the reduction of deleterious effects of the subsequent or previous damage. This resistance induction is a part of a general cellular response to stress that emerged in early evolution and has subsequently been observed in all organisms. The central hallmark of this induced resistance in prokaryotes is the ability to effectively repair DNA double-strand breaks, and this ability has been retained to a great extent through the evolution in cells of eukaryotes—unicellular eukaryotes, simple eukaryotes, insects, plants, amphibians, and mammals.

Great experience accumulated in the study of mechanisms of exogenous compounds on luminescent bacteria and their enzymatic reactions makes it possible to use these systems to study low-intensity exposure. The reviews by Girotti and Roda, University of Bologna, Italy, are considered to be classical [9,10,73]. In addition, in Russia marine bacteria are also used for this purpose, for example, at the Biology Department of Moscow State University [74], etc. The Gu group (National Environmental Biotechnology Research Laboratory, Kwangju, South Korea) was the first to apply bioluminescence to monitor the bioeffects of radiation (using gamma radiation as an example) [75]. In addition to the abovementioned groups, there are other studies which apply luminescent bacteria for monitoring the radiation bioeffects [6,72,76,77,78]. Unicellular bacterial responses form the basis for understanding the nature of low-dose effects on multicellular organisms.

Rozhko and coworkers [34], for the first time, applied luminous marine bacteria to monitor the low-dose effects of radionuclides; an alpha-emitting radioisotope americium-241 was chosen as an example in this study. Later, we explored the low-dose effects of alpha- (americium-241, uranium-(235 + 238); thorium-232) and beta-emitting radioisotopes (tritium) [31,78,79,80] as well as gamma-radiation [81]. The bacterial bioluminescence response to the radionuclides was demonstrated to include the following stages: (1) threshold, (2) activation, and (3) inhibition; and the combination of the stages can be different.

**Figure 2:**
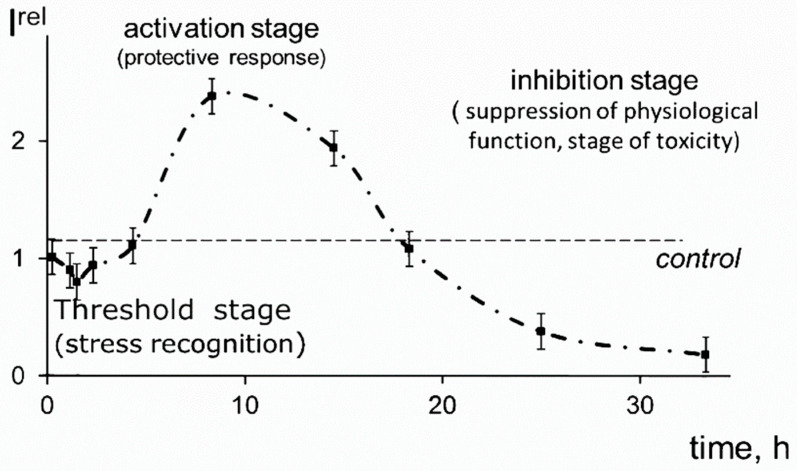
Bioluminescence kinetics of bacteria in the solution of americium-241, 3 kBq/L [72,80].

The activation of bacterial bioluminescence was observed in solutions of americium-241 (Figure 2), a radionuclide with high radioactive decay energy [6,34,80]. The bioluminescence kinetic curves in the solutions of americium-241 were fitted with the typical hormesis model, involving the activation stage.

It should be noted that the study of the bioeffects of americium-241 is highly valuable now. Americium-241 is a product of ^241^Pu radioactive decay; it is produced in nuclear power plants during the activation of ^239^Pu and ^240^Pu by neutrons, which is followed by the beta decay of ^241^Pu [82]. The environmental risk of contamination with americium-241 is due to the time-dependent increase of its concentration in the future, taking into account the long half-life of americium-241 (432 years). This problem will remain acute for many decades and centuries.

Americium-241 is known to be accumulated in the environment due to its ability to be bound by organic matter and to be concentrated on the cell surfaces as well as to penetrate through cellular membranes by means of siderophores—specific cellular proteins [83]. For example, the accumulation of americium-241 by the biomass of aquatic plants was found in the Chernobyl zone contaminated with radioactive fallout [84]. The accumulation of americium-241 by sediments, aquatic plants, and fish in the Siberian river Yenisei (Siberia, Russia) was under study in [85,86,87].

Alpha-emitting radionuclides thorium (Th) and uranium (U) are of particular interest, being the most abundant radioactive elements in natural ecosystems and important sources of nuclear energy [88,89,90,91]; there exist large areas contaminated with these radionuclides. They are longest-lived isotopes: Th-232 has a half-life of 14 × 10^9^ years; while U-235 and U-238 have half-lives of 7.04 × 10^8^ and 4.5 × 10^9^ years, respectively. Currently, the interest to thorium as an alternative source of nuclear energy is increasing, which can lead to an acceleration of its accumulation in the environment and expansion of areas with radioactive contamination [92,93].

However, the low-dose effects of thorium and uranium radioisotopes on organisms have not been thoroughly studied yet [94].

Rozhko and coworkers compared the effects of alpha-emitting radionuclides of different specific radioactivity, americium-241 and uranium-(235 + 238) on bacterial bioluminescence [95]. It was found that uranium-(235 + 238), a radionuclide with a low specific radioactivity, inhibited bacterial bioluminescence at concentrations >10^−5^ M (>30 Bq/L); activation of bioluminescence was not observed. The uranium effect was attributed to the chemical component of its impact rather than to the radioactive one.

**Figure 3:**
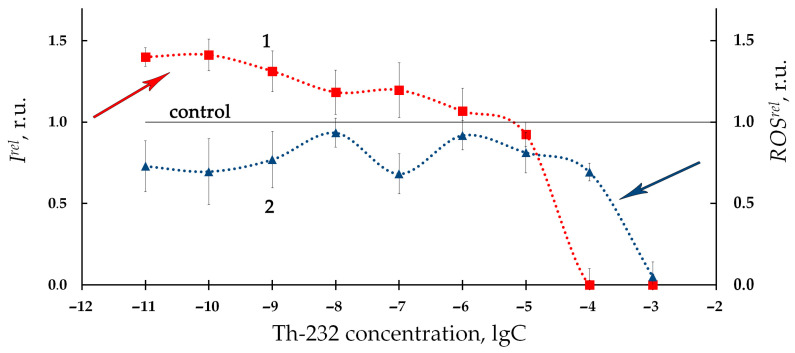
(1) Relative bioluminescence intensity, *I^rel^*, and (2) relative reactive oxygen species content, *ROS^rel^*, in a bacterial suspension at different concentrations of Th-232, M. The time of exposure to Th-232 was 1 h. The ROS content in the control (non-radioactive) sample was 5.5 × 10^–6^ M [96].

The study by Kolesnik et al. [96] was aimed at the bioeffect of the other low-active alpha-emitting radionuclide, thorium-232; intact bacteria and enzymatic reactions were applied as bioassays. Thorium nitrate, Th(NO_3_)_4_•4H_2_O, was used as a source of radiation; the dose accumulated in the bacterial culture did not exceed 0.1 Gy, which is below the conventional low-dose limit. Moderate bioluminescence activation (<50%) was found in the solutions of thorium-232. The low-concentration (10^−11^–10^−6^ M) activation of the bacterial bioluminescence is evident from Figure 3 (*I^rel^* > 1, red line). The bioluminescence inhibition (*I^rel^* < 1) at higher concentrations of thorium solutions (10^−6^–10^−3^ M) was attributed to pH shifts in bacterial suspensions rather than to the thorium radioactivity.

In a number of studies, we chose tritium as a model beta type radionuclide [79,97]. The choice of tritium was justified by its environmental occurrence due to its natural and anthropogenic origin: on the one hand, tritium is generated in the atmospheric top layers by space irradiation; on the other hand, it is a by-product of numerous nuclear industry processes. A local increase in the tritium content occurs around nuclear power plants, and it can rise dramatically after nuclear accidents. In terms of the chemical properties, tritium is similar to the stable hydrogen isotope and can replace it in organic compounds. Despite the low particle energy, tritium creates a significant density of ionization in solutions and tissues. As a result, the processes of charge transfer are intensified in the organisms, and the local impact of tritium decay can be quite strong.

The total energy of tritium beta-decay is low (18.6 keV), and the average energy of electrons is also low (5.7 keV). This is a reason for considering tritium as one of the less hazardous radioisotopes. The products of the tritium decay include an electron (a beta particle) and an ionized isotope of helium (H23e+):

H13→ β− H23e++e−+ν˜

**Figure 4:**
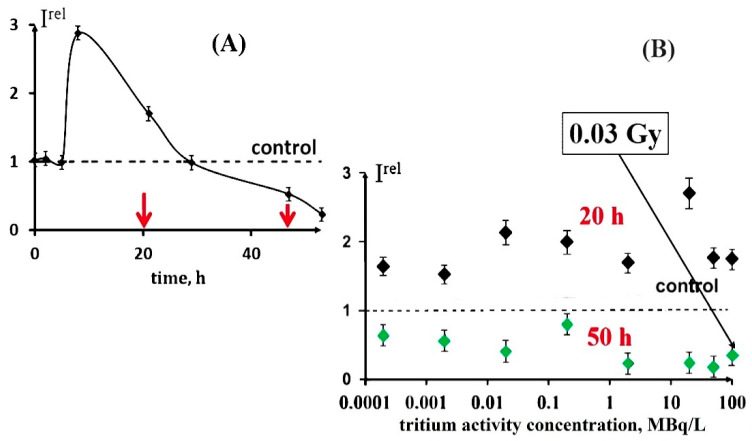
Effect of tritiated water on bacterial bioluminescence. (**A**) Bacterial bioluminescence kinetics in tritiated water, 2 MBq/L; the red arrows indicate the time of sampling (20 and 50 h); (**B**) bacterial bioluminescence intensity vs. activity concentration of tritiated water at different exposure times: 20 h (black) and 50 h (green) [72,78,98].

The products of the tritium radioactive decay are capable of triggering electron/charge transfer processes in biochemical reactions and they can affect the rates of cellular processes. The previous studies under chronic low-dose exposure (<0.03 Gy) [6,31,78,98] demonstrated both the inhibition and activation effects of tritium on luminous marine bacteria (Figure 4A), and the absence of any monotonic dependences of bioluminescence responses on the tritium concentration. These regularities were found in a wide range of tritium activity concentrations: from 0.0001 to 200 MBq/L (Figure 4B). This result can be explained in terms of the adaptation ability of the bacterial cells to the low-dose radiation, with the hormesis model involved. Additionally, Rozhko et al., 2019 showed that the bioluminescence intensity was the same for three activity concentrations of tritium (0.03 MBq/L; 4 MBq/L; 500 MBq/L) during the entire observation time (52 h) [79]. In this experiment, tritium increased the bioluminescence intensity by approximately three times as compared to the control.

An increase in the bacterial luminescence intensity in the presence of tritiated water, HTO, was demonstrated in a series of experiments. The bi-phasic dependence (activation + inhibition) was found in [31,80], while the mono-phasic dependence (activation only) was shown in [79,99,100].

Hence, the low-dose effects of tritium on the cellular level were shown to correspond to the model of ‘radiation hormesis’, including as they do the stages of activation and inhibition of physiological processes [6,29,78,97,98]. This fact demonstrates that the balance of aquatic biota in natural ecosystems under exposure to tritium can be shifted both to the activation and inhibition of the vital function of aquatic microorganisms, with further misbalance in food chains. 

Radiobiological effects of gamma irradiation are of high concern for researchers now, as a large number of people are exposed to radiation from various man-made and natural sources, particularly in the nuclear energy industry, in the course of conventional radiotherapy and diagnostic processes, including unintentional exposure to radiation [101,102]. Currently, gamma irradiation is one of the most promising and widely applied methods for disinfection of food products and plant materials [102].

**Figure 5:**
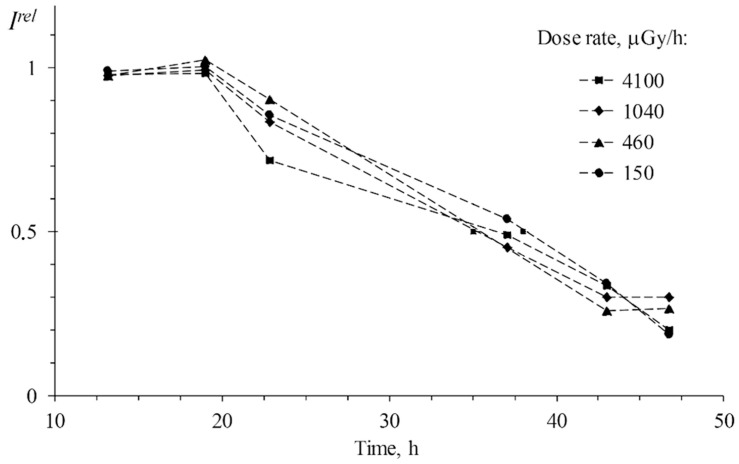
Bioluminescence intensity, *I^rel^*, of *P. phosphoreum* exposed to gamma radiation at different dose rates, ^137^Cs, 20 °C. The error for *I^rel^* was 10% [72,81].

Nevertheless, the effects of low-dose gamma-irradiation on luminous marine bacteria, a widely used bioassay system, have not been sufficiently explored. The study by Kudryasheva et al., 2017 [81] is an example of research in this field. The authors demonstrated the independence of the bacterial response on the irradiation intensity at the low-dose gamma irradiation (Figure 5), similar to the low-dose effect of tritium mentioned before. Figure 5 demonstrates the bacterial bioluminescence intensities at four different irradiation dose rates, with almost identical responses: no bioluminescence activation was found, with the kinetic curves corresponding to the ‘threshold’ model.

The experiments with low-dose gamma irradiation revealed another point: a lower temperature showed a lower sensitivity of the bacterial cells: unlike at 20 °C, the bioluminescence inhibition was not observed at 10 °C and 5 °C [81]. This result was explained by the temperature-dependent deceleration of metabolic processes in bacteria, including radiation-induced ones.

Hence, the independence of the bacterial bioluminescence response on the radiation intensity (the activity concentration or dose rate for alpha/beta radionuclides or gamma radiation, respectively) was experimentally demonstrated under the low dose exposures; however, the time dependence was evident, corresponding to the hormesis model (for the alpha/beta radionuclides americium-241/tritium) or to the threshold model (for gamma radiation). Therefore, one can suppose that the ‘cellular adaptive response’ can be related to both regularities: (1) independence of responses on the irradiation intensities and (2) non-linear time-dependent response of the hormesis/threshold type.

A question arises whether these two peculiarities are inherent to enzymatic reactions, i.e., biological systems of a lower level of organization. This question is concerned with molecular mechanisms, to be discussed in the next section.

## 3. Mechanisms of Low-Dose Effects on Bacterial Cells

Mechanisms of low-dose responses of bacteria can be considered at the biochemical, chemical, and physico-chemical levels. In this section, we review the radiation effects on enzymatic reactions, chemical low-molecular components of these reactions, and content of reactive oxygen species, respectively. Additionally, we briefly consider a possible role of DNA damage, which has poorly been considered for luminous bacteria. 

### 3.1. Changes in the Rates of Intracellular Enzymatic Processes under Exposure to Radionuclides

**Figure 6:**
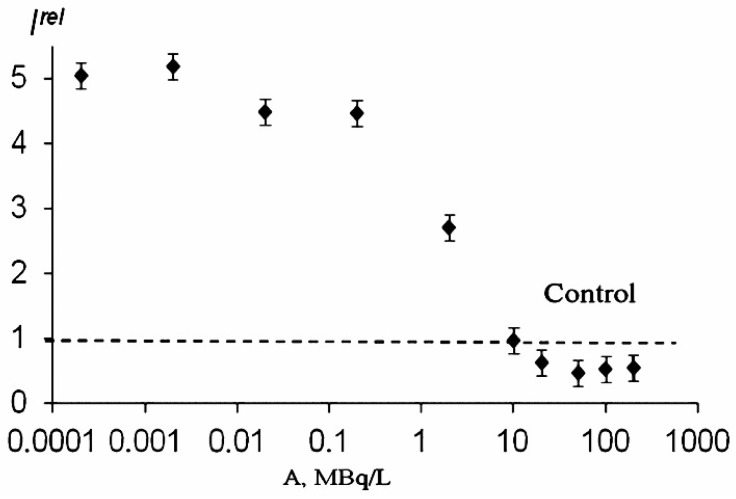
Bioluminescent intensity of the enzyme system, *I^rel^*, vs. radioactivity concentration of tritiated water, A, MBq/L [31,33,72,80].

The effects of alpha- and beta-emitting radionuclides (americium-241 and tritium) on the bioluminescence system of coupled enzyme reactions catalyzed by bacterial luciferase and NADH:FMN-oxidoreductase (see in Introduction) were studied in [31,33,80]. Bioluminescence activation and inhibition were observed. A monotonic dependence on the concentration of tritiated water is evident from Figure 6. However, the authors [78] did not find similar monotonic dependence in a wide concentration range of tritiated water, and bioluminescence activation was only registered within the range of tritium radioactivity concentration of 0.005–200 Mbq/L.

Similar to the luminous bacterial cells, a low-concentration increase in the bioluminescence intensity was observed in the enzymatic system under low-concentration exposure to thorium-232 [96], thus revealing the hormetic phenomenon in the enzymatic assay system in thorium solutions.

### 3.2. Consumption of An Intracecllular Reducer, NADH

NADH is an organic intracellular reducer; it can be considered an indicator of the reduction activity in enzymatic and cellular systems, which is involved into complex metabolic processes in organisms.

The rates of NADH oxidation were studied in solutions of the components of the bioluminescent enzyme system: the enzyme preparation and FMN [94]. The data obtained in this study are shown in Table 1. The rates of NADH oxidation were determined in the presence and absence of thorium-232.

An increase in the rates of NADH oxidation in the solutions exposed to thorium-232 can be found in all the samples (1–4, Table 1), with this increase being equal to 1.5–1.7. This result demonstrates that thorium-232 increases the efficiency of the reduction process involving enzymes and biologically important molecules (FMN and NADH). Hence, thorium can both (1) increase the bioluminescence intensity by accelerating the enzymatic processes and (2) decrease the bioluminescence intensity by accelerating the non-enzymatic processes and removing low-molecular components out of active enzymatic centers. The balance between these two processes depends on the peculiarities of the enzyme environment and these should be taken into consideration while explaining the activation or inhibition effects of radionuclides. 

### 3.3. Active Role of Reactive Oxygen Species

Molecular mechanisms of the radionuclide bioeffects are conventionally attributed to reactive oxygen species (ROS) which are generated in water bodies in the presence of dissolved molecular oxygen [31,88,103,104]. On the other hand, ROS are native products of metabolic oxidative processes in living organisms [105,106,107]. It was demonstrated that luminous marine bacteria naturally increase the ROS content in aquatic media, and intensify the ROS production upon the addition of tritium [78,97].

Chemically, ROS are products of the partial reduction of oxygen; the ROS group includes hydroxyl radicals (OH•), hydrogen peroxide (H_2_O_2_), superoxide anion (O_2_•−), etc. [102]. 

According to modern approaches, ROS are able to produce both damaging and signal bioeffects [108,109]; they regulate vital functions, such as cellular protective or apoptosis responses [103]. ROS are responsible for migration, proliferation, and differentiation [110,111]; they are known as stimulators of cell division [112,113] and cell death—apoptosis, necrosis, and autophagy [114,115]. The signal function of ROS is now being discussed [112,116,117,118]. It should be noted that ROS can serve both as inter- and intra-cellular messengers [119,120,121]. Both reactive oxygen and nitrogen species [122] released by cells can serve as signal particles which initiate the radiation-induced ‘bystander effect’ [123,124]. It is stated in [125,126,127] that ROS are responsible for both inhibiting (toxic) and activating bioeffects. It is noted in the papers mentioned before that the lack of ROS can suppress biological functions, similarly to the excess of ROS, but only the latter is widely and conventionally stated and discussed in biomedical literature. The reason for both effects is the disturbance of the ROS balance in bacterial suspensions.

Rozhko et al. [79] explained the decrease in the ROS content in bacterial suspensions in tritiated water by consumption of ROS in the bacterial bioluminescence reaction followed by the formation of a reaction intermediate–peroxide flavin derivative [21,128]. An increase in the ROS content was also observed in this study, which was explained by the intensification of complex metabolic processes in bacterial cells under radioactive exposure to tritiated water, similarly to the explanation presented in [79]. Direct correlations between the time-dependences of the ROS content and the bacterial bioluminescence intensity were found in the studies by Rozhko [79,97], presenting the basis for the explanation of the bioluminescence activation or inhibition under exposure to the radionuclide.

The luminescent marine bacteria naturally increased the ROS content in aqueous media, and additionally increased the ROS production up to 300% in the presence of tritium [79]. Hašler et al. [129] confirmed that tritiated water can stimulate the ROS production in another type of bacteria, *Pseudendoclonium basilense*, a bacterial strain from standing water. The 300% activation of luminescence of marine bacteria by tritiated water was attributed to the ‘bystander effect’ [79]. The result was explained by the ‘trigger’ effect of tritium decay products, and by the signaling function of ROS.

**Figure 7:**
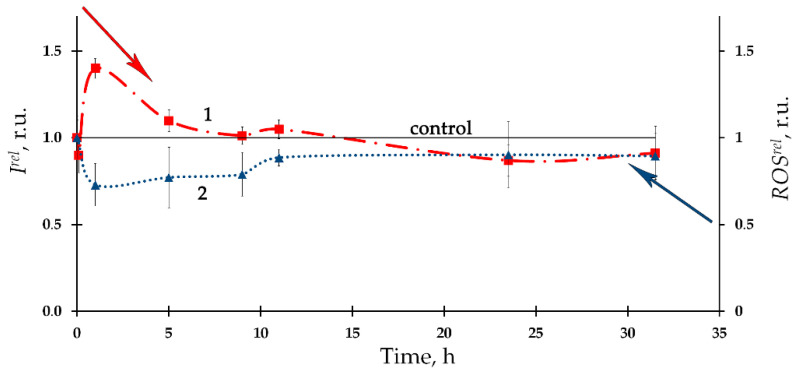
(1) Kinetics of bacterial bioluminescence, *I^rel^*, and (2) ROS content, *ROS^rel^*, in the presence of thorium-232, 10^−7^ M [96].

The time-course of the ROS content in the bacterial suspension in the presence of the alpha-emitting radionuclide thorium-232 is presented in Figure 7 as curve 2, according to [96]. A moderate decrease in the ROS content (compared to the nonradioactive control sample) with a tendency to its restoration was found. Negative correlations between the ROS content and the bioluminescence intensity were found in this study, thus demonstrating inverse relations between the bacterial physiological functions and the ROS concentration in the environment. It was concluded that the consumption of ROS contributes to the bioluminescence activation under low-dose exposure to thorium-232. It is assumed that the role of ROS should be taken into consideration in studying molecular mechanisms of the ‘hormesis’ approach [53,130,131,132].

Hence, one should indicate the differences in the correlations for the effects of the alpha and beta emitting radionuclides (americium-241 and tritium, respectively): correlation coefficients between the time-dependences of the bioluminescence intensity and the ROS content differed in their sign. This fact reflects the complexity of the ROS-dependent processes occurring in the biological systems under exposure to radionuclides of different types. 

### 3.4. Repair of DNA Damage

The first hypothetical mechanism of the hormesis phenomenon is based on repairing DNA damage [75,133,134]. The involvement of non-genetic mechanisms into low-dose chronic radioactive effects in luminous bacteria was proved earlier by Rozhko et al. [99,100] with the reference to the activation of membrane processes as a result of ionization of water media in radioactive solutions [7,135]. 

## 4. Role of Humic Substances as Radioprotective Agents in Water Solutions

Humic substances (HS) are complex mixtures of natural origin, being high-molecular organic compounds. They are formed due to the decomposition of animal and plant residues by microorganisms or due to abiotic environmental impacts [136]. HS can be found in soil, lakes and rivers [137]; they are included into and can be produced from peat, coal, sediments, and solid fossil fuels. The HS content in soil and natural water bodies is about 60–80% of the total organic matter, while in peat and coal, their content ranges from 20 to 90% [138].

Studying the effects of low-intensity radiation in the presence of HS forms the basis for predicting the response of living organisms to radiation in large areas contaminated with radionuclides after accidents, discharges from nuclear power plants or underground mining of natural resources [30,32,44,49,50,139].

**Figure 8:**
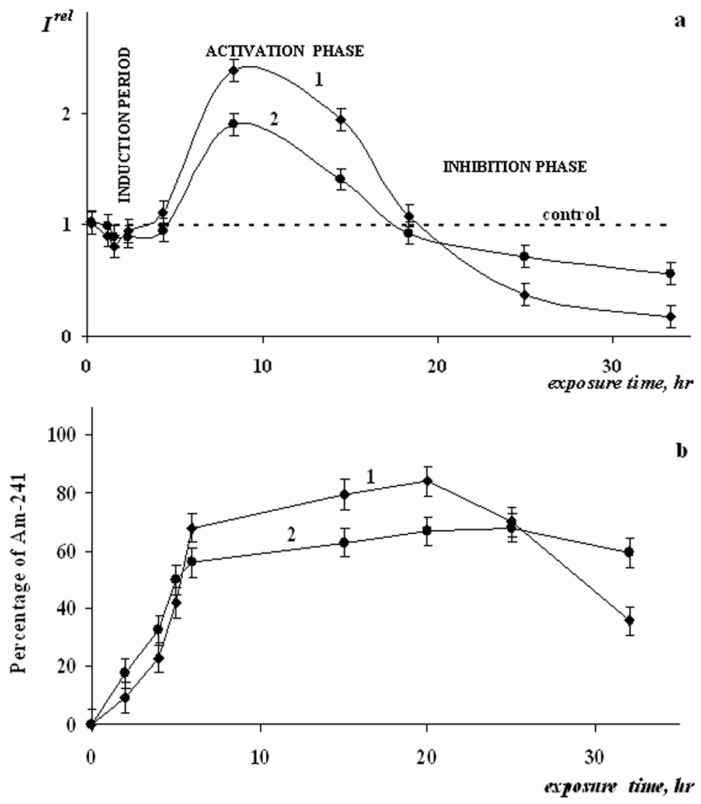
Chronic effects of Am-241 on luminous bacteria, (**a**) kinetics of the bioluminescence intensity, *I^rel^*, (**b**) percentage of Am-241 in the cellular fraction: (1) in the absence of humic substances; (2) in the presence of humic substances. The radioactivity of Am-241 solution was 3000 Bq/L (C  =  10^−10^ M). The concentration of the humic substances was 0.25 g/L [50,140].

Rozhko et al. and Kamnev et al. [140,141] analyzed the radioprotective effects of HS on luminescent marine bacteria exposed to the alpha-emitting radionuclide americium-241. Figure 8 shows the bacterial luminescence kinetics in an americium-241 solution in the absence and presence of HS. It evident that HS bring the kinetic curve closer to the control one, thus mitigating the effect of the radionuclide on the bacteria.

**Figure 9:**
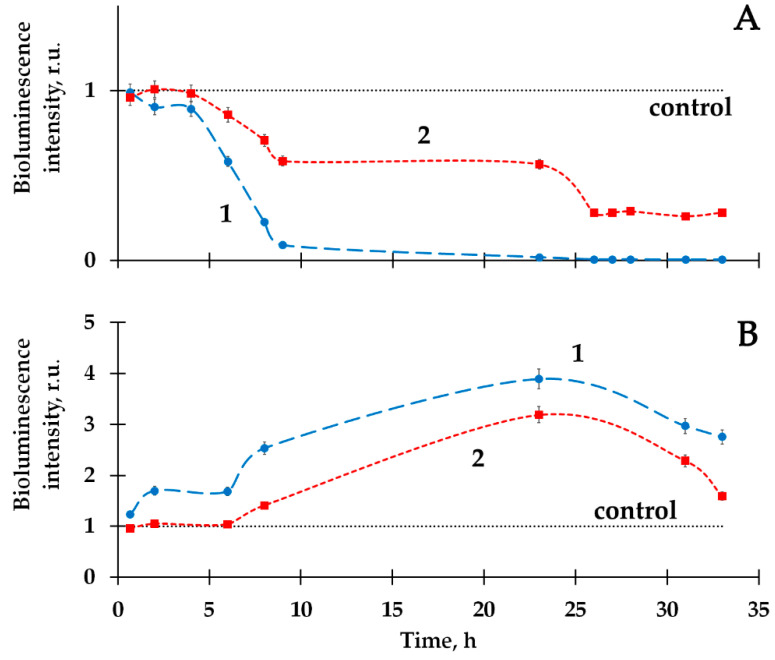
Bacterial bioluminescence kinetics in HTO in the absence (1) and presence (2) of humic substances (HS). The specific radioactivity of HTO: (**A**) 2 MBq/L; (**B**) 50 MBq/L. The HS concentration was 10^−3^ g/L [97].

HS were shown to serve as radioprotectors in the solutions of the beta-emitting radionuclide tritium; they were found to reduce the inhibitory and activating effects of tritium, similarly to those of americium-241 (Figure 9, curve 2) [97].

Correlations between the ROS content and the bioluminescence yield were found by Rozhko, Kolesnik et al. [97], confirming the ROS involvement in the low-dose effects of HS and tritium.

No significant protective ability of HS was found in solutions of the alpha-emitting radionuclide with low specific radioactivity, namely uranium-(235 + 238) [142]. Solutions of another alpha-emitting radionuclide thorium-232 were moderately detoxified by HS, with the HS detoxifying ability depending on the exposure time, not exceeding 35% [143]. The studies mentioned [95,137,138,140] used the Gumat-80 preparation, ‘Gumat’, produced by non-extracting treatment of coal in Irkutsk, Russia. The characteristics of the preparation were following: humic acids ≈ 85%, soluble potassium—9%, iron—1%, water—5%, pH 8–9 in a 1% water solution. The variation of the HS types from different natural sources could probably lead to various quantitative radioprotective characteristics of HS.

The model experiments with marine bacteria discussed in the present section show that the humic substances serve as radioprotectors in natural water bodies in the presence of radionuclides. However, there exists evidence that other substances with an antiradical feature [144,145] also provide the radioprotective function. Investigation of the radioprotective ability of different substances, involving carbon nanoparticles with unsaturated bonds, for example, series of fullerenes of different structure, is a highly promising field which can provide ecological industry with effective radioprotectors.

## 5. Conclusions

This review summarizes the studies of marine microorganisms under the conditions of low-dose irradiation of alpha, beta, and gamma types. Luminous marine bacteria were chosen as an example of such microorganisms since they are known to be sensitive to toxic compounds. Bioluminescence intensity was used here as the main physiological test parameter of the bacteria. The aspects of molecular intracellular processes under exposure to low-intensity radiation were analyzed, namely: changes in the rates of enzymatic processes in the bacteria, consumption of an intracellular reducer, NADH, active role of reactive oxygen species, and repair of DNA damage. 

Three stages in the bioluminescent response to radionuclides were found under the conditions of low-dose irradiation: (1) absence of effects, (2) activation, and (3) inhibition. The activation of bacterial bioluminescence was observed in the solutions of alpha-emitting radionuclides, americium-241 and thorium-232. An increase in the bacterial luminescence intensity in the presence of beta-emitting radionuclide tritium (in tritiated water, HTO), was also found. 

The ability of HS to protect microorganisms in the solutions of alpha- and beta-emitting radionuclides, americium-241 and tritium, was reviewed.

The results can contribute to the understanding of the perspectives of bioluminescent assays for monitoring low-intensity radioactive exposures. Marine bacteria could be considered as a simplified model for understanding the effects of low-dose radiation on higher organisms.

## Figures and Tables

**Table 1:** Rates of NADH oxidation (V) in the solutions of different composition. The wavelength of optical density registration was 340 nm. The concentration of Th(NO_3_)_4,_ was 10^−7^ M [96].

Number of Solutions	Components of Solutions	V∙10^8^, M	
		without Th	with Th
1	NADH	2.43	4.05
2	NADH + enzyme preparation	4.05	6.07
3	NADH + FMN	14.20	20.60
4	NADH + FMN + enzyme preparation	16.20	26.70

## Data Availability

Not applicable.

## Abbreviations

FMN	flavin mononucleotide
HS	humic substances
HTO	tritiated water
NADH	nicotinamide adenine dinucleotide
ROS	reactive oxygen species
